# Fungi and insects compensate for lost vertebrate seed predation in an experimentally defaunated tropical forest

**DOI:** 10.1038/s41467-021-21978-8

**Published:** 2021-03-12

**Authors:** Peter Jeffrey Williams, Robert C. Ong, Jedediah F. Brodie, Matthew Scott Luskin

**Affiliations:** 1 Division of Biological Sciences, University of Montana, 32 Campus Drive, Missoula, MT 59812 USA; 2 Sabah Forestry Department, Forest Research Centre, Sepilok, P.O. Box 1407, 90715 Sandakan, Sabah Malaysia; 3 Wildlife Biology Program, University of Montana, 32 Campus Drive, Missoula, MT 59812 USA; 4 School of Biological Sciences, University of Queensland, 534 Goddard Hall, St. Lucia, QLD Australia

**Keywords:** Community ecology, Tropical ecology

## Abstract

Overhunting reduces important plant-animal interactions such as vertebrate seed dispersal and seed predation, thereby altering plant regeneration and even above-ground biomass. It remains unclear, however, if non-hunted species can compensate for lost vertebrates in defaunated ecosystems. We use a nested exclusion experiment to isolate the effects of different seed enemies in a Bornean rainforest. In four of five tree species, vertebrates kill many seeds (13–66%). Nonetheless, when large mammals are excluded, seed mortality from insects and fungi fully compensates for the lost vertebrate predation, such that defaunation has no effect on seedling establishment. The switch from seed predation by generalist vertebrates to specialist insects and fungi in defaunated systems may alter Janzen–Connell effects and density-dependence in plants. Previous work using simulation models to explore how lost seed dispersal will affect tree species composition and carbon storage may require reevaluation in the context of functional redundancy within complex species interactions networks.

## Abstract (web summary)

Ecologists predict that loss of large vertebrates will alter tropical plant communities. Here, the authors report a field experiment on seed mortality and seedling establishment in Borneo, in which experimental defaunation of large seed consumers was functionally compensated by insects and fungi.

## Introduction

Hunting is reducing large vertebrate populations around the world, a phenomenon known as defaunation^1^. This can have cascading impacts due to altered plant-animal interactions^1–3^, with potential consequences for plant species composition, forest regeneration, and even ecosystem functions such as carbon storage^4–6^. For example, defaunation-induced losses of seed dispersal by large vertebrates have been predicted to drive declines in the largest and heaviest-wooded tree species—many of which are vertebrate dispersed—decreasing above-ground biomass and carbon storage by 2.4–26% in the Amazon^7^, Brazilian Atlantic Forest^8^, and Thailand^9^. However, reduced dispersal only causes plant population declines if there is strong conspecific negative distance- or density-dependent mortality for undispersed seeds, which is lacking in many plant species^10,11^. Furthermore, even assuming that defaunation-caused dispersal limitation increases seed mortality, this could be offset by the concurrent decline in large vertebrate seed predation^12^. Therefore, predicting the cascading impacts of defaunation on plants requires understanding the effects of both altered seed dispersal and seed predation^8^.

Seed predation is a key interaction that strongly influences plant abundance^13,14^, coexistence^15,16^, and diversity^17,18^. Seed predation in tropical forests is largely performed by insects^19,20^, fungi^21,22^, and vertebrates^14,23,24^. The Janzen–Connell hypothesis suggests that host-specific insect and fungal enemies cause conspecific density-dependent mortality for under-dispersed offspring, which promotes species coexistence and diversity^25–28^. However, different seed predators cause different spatial patterns of mortality, depending on their mobility and diet specificity^11,28,29^. Importantly, wide-ranging generalist seed predators, such as most vertebrates, are comparatively less important sources of Janzen–Connell effects^11,28,30^. Therefore, if defaunation switches the dominant seed predators from non-selective vertebrates that do not induce density-dependence to fungi and insects that do induce density-dependence, this could alter species composition, coexistence, and diversity. To date, studies have observed that defaunation can change patterns of vertebrate seed predation^31–33^, but the net effects on seed survival remain largely unknown because there is little information on how other non-hunted seed enemies respond to defaunation.

Here we assess whether fungi, insects, or smaller vertebrates compensate for the loss of the larger hunted vertebrate seed predators. If the different enemy groups are functionally redundant, then reduced seed predation by larger animals in hunted forests could be compensated by increasing populations or feeding rates of other enemies, leading to no net change in seed survival or seedling recruitment^34^. Alternatively, seed predators could have additive effects, meaning that the loss of one species or group may not be compensated by others. For example, if seed predators are specialized to attack different species, types, or sizes of seeds, they may be unable to expand their diet breadths (in an ecologically relevant timeframe) to predate unexploited seed resources^34^. If seed predator effects are additive, then seed survival and seedling recruitment could increase in defaunated forests, in which case higher survival due to lost vertebrate seed predators could potentially offset the negative effects of reduced zoochorous seed dispersal on plant recruitment^12^.

There is evidence that hunted tropical forests can experience changes in plant recruitment, but the directionality is inconsistent. Defaunation has been associated with declining seed predation and higher plant recruitment^35–37^, but also compensatory rises of seed-predating rodent populations leading to lower plant recruitment^38–40^. In one study in the Neotropics, insects partially compensated for reduced vertebrate seed predation in overhunted forests^19^. The role of compensatory seed predation by both insects and fungi remains largely untested. Collectively, the degree to which the effects of different seed predator groups are predominately additive or compensatory remains unclear, limiting our understanding of how defaunation will alter plant regeneration and influence Janzen-Connell effects.

**Fig. 1:**
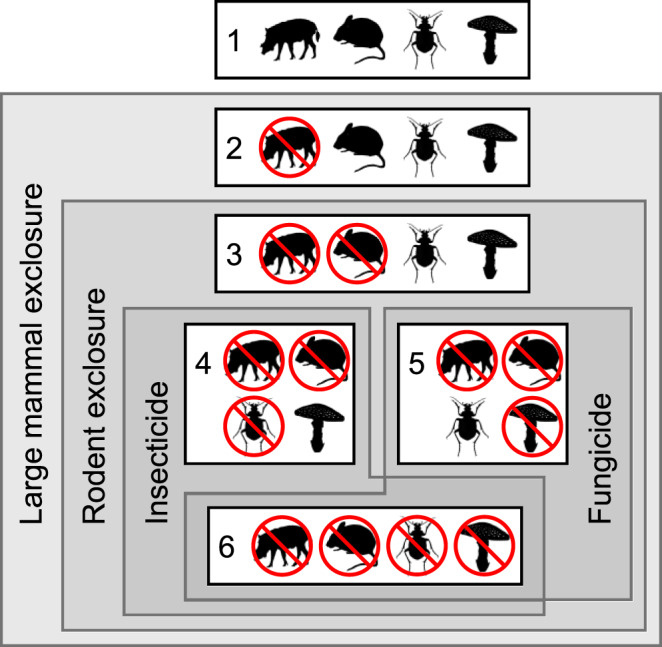
Experimental design. Diagram of the nested exclusion experimental design used here with 10 replicate blocks, each containing the 6 treatments shown.

**Fig. 2:**
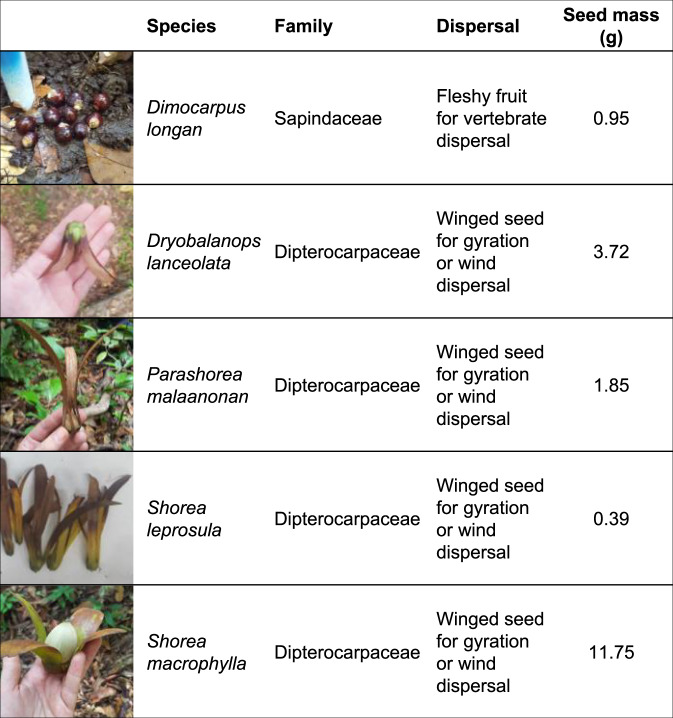
Species used in experiment. Information on the five tree species used in the nested exclusion experiment here.

We experimentally test whether other seed enemies compensate for lost seed predation by large vertebrates in a nearly faunally intact lowland rainforest in Borneo. We use a nested set of treatments to experimentally isolate the effects of large vertebrates, small vertebrates, insects, and fungi on seed survival (Fig. 1). Specifically, we disentangle predation by large terrestrial vertebrates (>~1 kg) using fenced exclosures and by rodents using smaller wire cages, and we attributed seed mortality to vertebrates based on physical signs (such as chew marks) or seed removal (see Methods, below). We established insecticide and fungicide treatments within the vertebrate exclosures. Our study includes seeds from five native tree species, including four members of the dominant family Dipterocarpaceae and one species (*Dimocarpus longan*), that is native in the region and also cultivated for its fleshy fruit (Fig. 2).

Our results show that large hunted vertebrates are important seed predators, but we observe strong compensatory increases in seed mortality from insects and fungi when large vertebrates are excluded. This suggests that the defaunation-induced loss of vertebrate seed predation does not affect seed survival. If our results are consistent across the tree community, there would be no reduction of seed mortality to offset the negative effects of defaunation-caused dispersal limitation for zoochorous plants.

## Results

### Fungal and insect enemies compensate for vertebrate seed predation

**Fig. 3:**
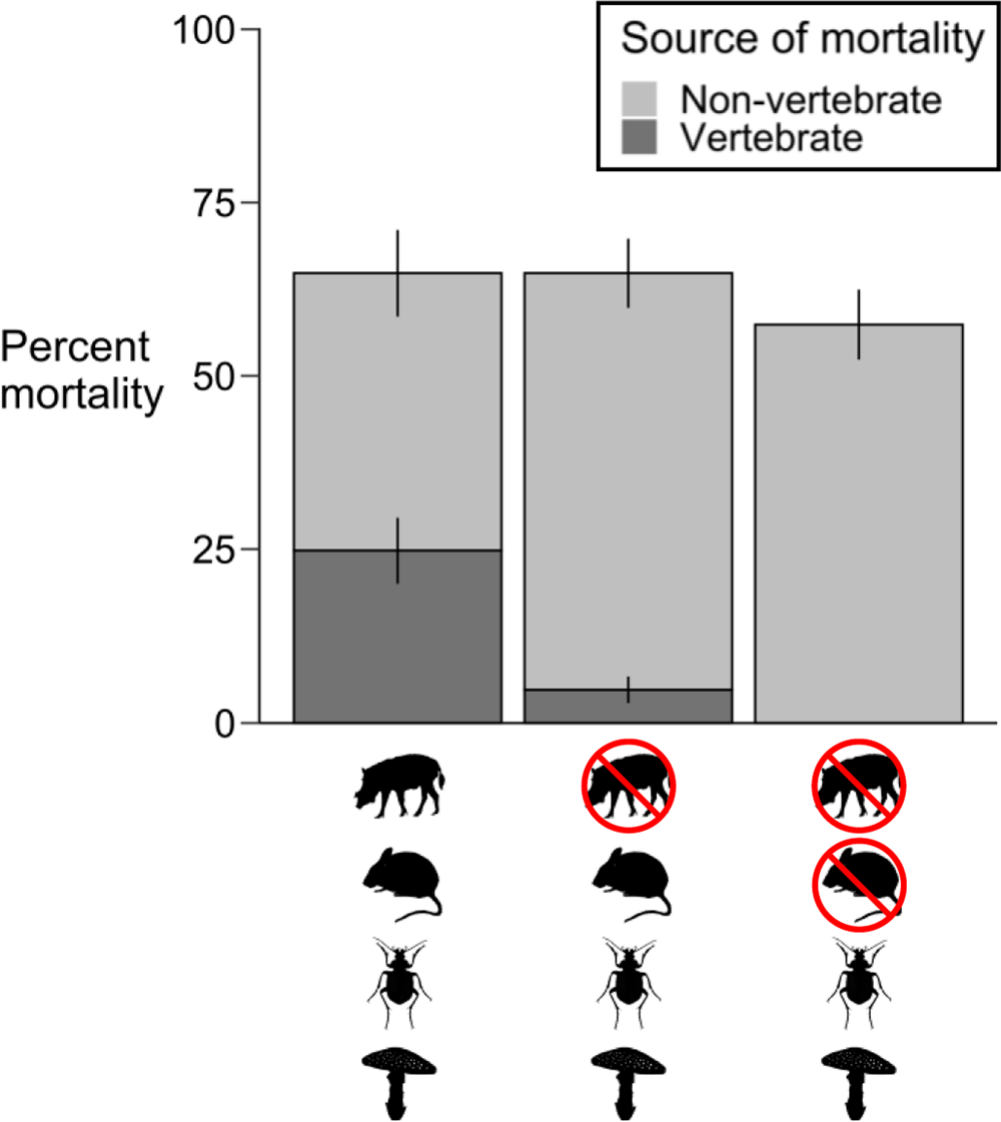
Source of seed mortality. Total seed mortality did not differ based on the presence or absence of large vertebrates (mixed-effects logistic regression: *P* = 0.887), even though vertebrate mortality significantly declined when large animals were excluded (*P* < 0.001), due to compensatory predation by fungi and insects. Total mortality also did not change when small mammals were excluded (*P* = 0.411). Mortality is defined as seeds that died prior to establishing as seedlings and was calculated across all species (*N* = 500 seeds per treatment for each of the five species). Error bars show mean values ± standard errors across experimental blocks (*N* = 10).

**Fig. 4:**
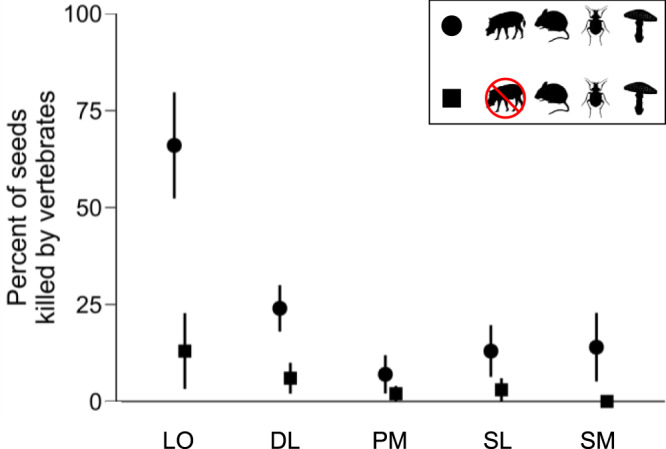
Seed predation. Differences in seed predation by large and small vertebrates (circles) vs. small vertebrates only (squares); error bars show mean values ± standard errors across experimental blocks (*N* = 10). Predation was determined by physical signs or seed removal and limited to the period prior to establishing as seedlings (i.e., excluding trampling). LO = *Dimocarpus longan*, DL = *Dryobalanops lanceolata*, PM = *Parashorea malaanonan*, SL = *Shorea leprosula*, and SM = *Shorea macrophylla*. Excluding large mammals significantly decreased vertebrate predation (mixed-effects logistic regression: *P* < 0.001). Overall vertebrate mortality did not significantly differ only for PM (*P* = 0.083). This suggests that large vertebrates are significant seed predators for LO, DL, SL, and SM.

**Fig. 5:**
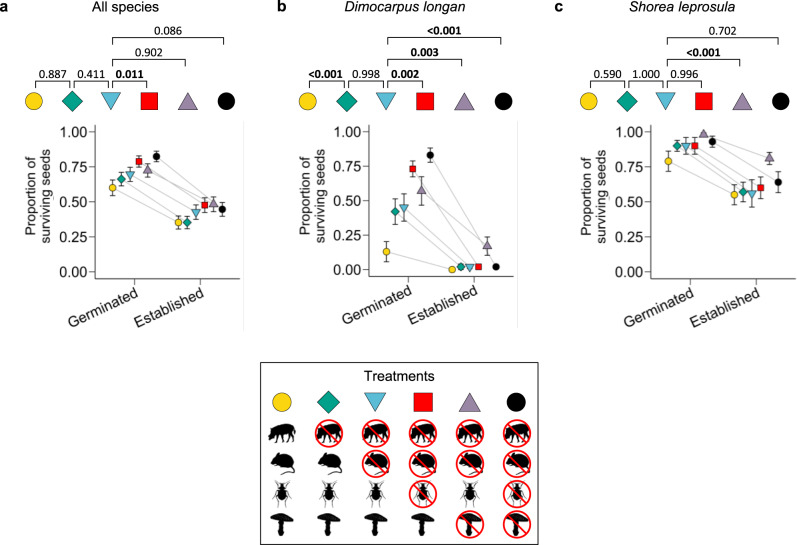
Seed survival. Proportions of seeds surviving under experimental treatments (indicated by different colors and shapes); error bars indicate mean values ± standard errors across treatments (*N* = 50 for all species; *N* = 10 for individual species). Logistic regressions were performed to test the effect of treatment on seed survival. Then, post-hoc Tukey tests were used to adjust for multiple tests and identify significant pairwise comparisons. All statistical tests were two-tailed. Selected pairwise comparisons shown with P-values; significant comparisons in bold. **a** Across species, insecticide increased survival (*P* = 0.011). **b**,**c** Fungicide increased survival in *Dimocarpus longan* (*P* = 0.003) and *Shorea leprosula* (*P* < 0.001).

Across all tree species, vertebrate seed predation significantly declined inside large-vertebrate exclosures (4.8% of seeds predated) as compared to outside the exclosures (24.8% predated; mixed-effects logistic regression: β = -1.89 ± SE = 0.47, *P* < 0.001; Fig. 3), suggesting that large vertebrates were significant seed predators. The exclusion of large vertebrates reduced vertebrate seed predation in all five species, though for one species this reduction was not significant (see Species-specific results, below; Fig. 4). Despite reduced vertebrate seed predation inside fenced exclosures, overall seed survival did not differ between the exclosures and open control plots (β = 0.30 ± 0.30, *P* = 0.887; Figs. 3 and 5), indicating compensatory seed predation by other enemies (i.e., fungi and insects). We confirmed that insects and fungi were significant non-vertebrate seed enemies because applying insecticide increased seed survival across all species when the taxa were analyzed together (β = 0.53 ± 0.16, *P* = 0.011; Fig. 5a), and applying fungicide increased seed survival for two of the five species individually (see below; Fig. 5). Surprisingly, small vertebrates killed few seeds of any species, and excluding them did not significantly increase seed survival when all species were analyzed together (β = 0.37 ± 0.21, *P* = 0.411; Figs. 3, 4, 5).

### Species-specific results

In models with each species analyzed separately, we found that excluding large vertebrates significantly reduced vertebrate predation in four of our five species (P < 0.001 in all cases) but not in *Parashorea malaanonan* (β = -1.51 ± 0.87, *P* = 0.083, Fig. 4). Large vertebrates ate too few *Parashorea malaanonan* seeds so there was not an opportunity for compensation in this species.

Fungicide did not increase seed survival across all species combined (see Fungal and insect enemies compensate for vertebrate seed predation, above), but it did increase survival for *Shorea leprosula* (β = 1.39 ± 0.31, *P* < 0.001, Fig. 5c) and *Dimocarpus longan* (*β* = 1.03 ± 0.28, *P* = 0.003, Fig. 5b) individually. Excluding large vertebrates increased *Dimocarpus longan* seed survival to the germination stage (β = 1.70 ± 0.36, *P* < 0.001), but survival to seedling establishment was unaffected by experimental defaunation (Fig. 5b).

## Discussion

We observed strong compensation among seed predator groups, and this has important conservation implications given the widespread destruction of large-bodied granivorous vertebrates globally^41^. Large mammals were significant seed predators in our system, but their exclusion had no effect on overall seed survival because—when they were removed—predation by fungi and insects fully compensated. This suggests that seed enemies are predominately redundant, rather than complementary, in this system. If such patterns hold at the community-scale, abiotically dispersed trees are unlikely to be affected by defaunation because overall seed predation may remain constant. For zoochorous trees, the negative effects of hunting-induced dispersal limitation^42,43^ may not be offset by the positive effects of reduced vertebrate seed predation.

A key insight from our findings is the potential for defaunated systems to switch from seed predation patterns dominated by generalist vertebrates to seed predation by specialist insects and fungi. The switch towards higher seed mortality from insects and fungi may have important implications for tree composition by altering Janzen–Connell effects and conspecific negative density-dependence. Janzen–Connell effects are driven by host-specific and dispersal-limited insects or fungi enemies^28,29^, which cause higher seed and seedling mortality nearby conspecific adults^11^. However, vertebrate generalists consume many plant species and are highly mobile, making them unlikely sources of conspecific negative density-dependence^28^. Therefore, a switch towards increased seed mortality from insects and fungi in defaunated forests could potentially increase conspecific negative density dependence and drive patterns of diversity and coexistence^27,28^.

Rodent seed predation appears to be relatively unimportant in our system, which contrasts with observations from other work^19,38,40^. This may be because rodents in defaunated forests can increase in abundance (e.g., following release from predators and competitors)^38^, which may then compensate or overcompensate for the lost seed predation by larger-bodied granivores^38–40^. Our large-vertebrate exclosures (99 m^2^ each) were too small to trigger population-level increases in rodents. Rodents tend to predate smaller seeds than those consumed by larger mammals^44,45^. It has remained unclear whether rodents would shift their diet breadths once their larger competitors were eliminated^32,46,47^, though our study suggests that this might not occur.

The net effects of defaunation on plants will vary depending on whether the plant species is vertebrate-dispersed and has seeds that are targeted by vertebrate predators^33^. Seed predation is clearly more important than seed dispersal for abiotically dispersed species such as the dipterocarps that dominate the canopies of Southeast Asian equatorial forests. Our study—which included four dipterocarps—suggests that defaunation might not affect these species’ populations. This conclusion is consistent with prior modeling work^6^. For vertebrate-dispersed trees, reduced seed dispersal from defaunation may still lead to shifts in species composition and even the erosion of forest carbon storage^4,6,7,9^ if lost seed dispersal by large vertebrates is not compensated for by the remaining species in the community. But overall, it remains unclear how defaunation will affect vertebrate-dispersed tree species given the complex, concurrent impacts on seed predation.

In one of the largest empirical studies of the cascading impacts of defaunation on tropical tree communities, Harrison et al.^37^ found that spatial aggregation increased for vertebrate-dispersed trees but not for abiotically-dispersed species, driven by the loss of dispersers such as primates and hornbills. However, they also found that sapling abundances increased >25% over the two-decade study period, which is the opposite of what would be predicted if undispersed seeds tend to suffer high conspecific negative density-dependence. Harrison et al.^37^ suggest that the higher sapling recruitment was due to the absence of seed predators and herbivores. But our results show that compensatory effects of other enemies may have offset the effects of lost seed predators. Therefore, we suspect that the explanation for enhanced sapling recruitment was lower seedling herbivory as well as non-trophic effects of megafauna such as trampling seedlings that have already established. The importance of non-trophic disturbances on seedling and sapling mortality is supported by two recent studies. First, a study monitoring artificial seedlings found that wildlife may trample >50% of stems in Malaysian Borneo^40^. Second, in a Peninsular Malaysian primary forest where wild pig (*Sus scrofa*) populations are elevated due to food subsidies from crop-raiding in adjacent oil palm plantations, pig nest construction was the primary driver of a 62% decline in saplings over a two-decade study period^48^. There appears to be an immense effect of trampling and nest building on seedlings in Malaysian forests, and this could explain why Harrison et al.^37^ observed higher sapling recruitment in a defaunated Malaysian forest even in the presence of compensatory seed predation.

Generalizations from our findings are limited by the relatively few species that we assessed and the short duration of our study. We focused on only five species in two families, out of >700 species at our site, and our study was undertaken in a single season. Therefore, we do not know how widespread compensatory seed predation is in our tree community or in other systems. Our site also lacks a persistent seedbank, which is true of most tropical rainforest tree communities^49^, but this limits the extrapolation of our results to temperate systems where longer-lived seeds face different predation pressures than those that germinate quickly^50^. Finally, we did not conduct full population-level demographic analyses, so we are not able to assess how our measured impacts on seed predation would translate into impacts on plant abundance. Nevertheless, early life-stage vital rates generally have very low elasticities in long-lived tree species^51^, so even relatively large changes in seed predation often have limited impact on plant abundance^52^ (and we observed no defaunation-induced changes in overall seed predation at all).

Seed traits such as size and toxicity likely influence which enemies attack seeds. By altering seed predator assemblages, defaunation could affect which seeds are consumed. For example, Mendoza and Dirzo^32^ hypothesized that larger seeds escape rodent granivory due to poor handling efficiencies and so would experience less seed predation in defaunated areas. We did not see any effect of seed size in our study, though our sample size was too small to explicitly examine this trait, and a meta-analysis did not find a correlation between seed size and changes in seed predation under defaunation^31^. We found the insects had stronger effects on seed survival than fungi in defaunation treatments. This is consistent with results from a recent greenhouse experiment that found very weak effects of fungal pathogens on dipterocarp seedling mortality overall^53^. In fact, that study included three of the dipterocarp species we used, and their fungicide results were non-significant for *Dryobalanops lanceolata* and *Parashorea malaanonan* and significant for *Shorea leprosula*, which is exactly the pattern we observed in our field experiment. Other studies suggest that seed-predating insects are resistant to disturbances such as hunting and logging^54,55^, though defaunation could have cascading effects on seed-predating insect communities^56^. Compensation by insects and fungi may be mediated by seed traits, but the field currently lacks sufficient data to assess how defaunation will affect seed mortality across plant traits.

Defaunation will alter tropical tree communities, but we require more empirical work to unravel the complex interactions that determine the net impacts on plant recruitment. Future research should focus on expanding the set of tree species tested at a single site to determine if the patterns we observed for five tree species are representative of a community-wide phenomenon. In lieu of testing hundreds or thousands of species, studies that focus on how plant traits affect susceptibility to specific enemy guilds could also help in developing inference about community-wide patterns. We caution that previous research assuming that losses in seed dispersal will lead to population declines may be premature, since reduced seed predation may offset this^12^, or, as we show, compensatory increases in other seed predator guilds can shift which predators are important in a system. We are especially interested in whether the compensatory increase in insect and fungal seed predation in defaunated forests alters density-dependence, as this could interact with altered seed dispersal^7,42^ and affect species coexistence and diversity^27,28^. Despite a significant body of work investigating the cascading impacts of defaunation on plant communities, there remain more questions than answers; this is an applied ecological issue that is ripe for new discoveries.

## Methods

### Study system

We conducted our research in the lowland dipterocarp forests of Danum Valley Conservation Area in the state of Sabah, Malaysian Borneo. The majority of the conservation area consists of terrain below 700 m elevation and has no history of logging^57^. Danum Valley has a wet equatorial climate, with ~2800 mm of annual rainfall, a mean maximum temperature of ~31 °C, and a mean minimum temperature of ~23 °C^57^. The forests are dominated by trees in the Dipterocarpaceae family with a canopy ~60 m high and even taller emergent trees^58^. Dipterocarp trees produce non-fleshy, lipid-rich fruits that are abiotically dispersed by wind and gyration and predated by many vertebrates^24^. A large percentage of Borneo’s lowland tree species produce flowers and seeds in irregular, supra-annual masting cycles, with dipterocarps showing particularly strict adherence to this pattern^59^. Major post-dispersal seed predators of large tree seeds in this system include bearded pigs (*Sus barbatus*), porcupines, murid rodents, and insects such as beetles^24,60^. Other large terrestrial vertebrates that may consume tree seeds include sun bears (*Helarctos malayanus*)^61^, pheasants^62^, several ungulate species (sambar, muntjac, chevrotain)^63^, and several primate species (macaques and orangutan)^62^. This area is faunally intact, with the exception of the Sumatran rhinoceros, *Dicerorhinus sumatrensis*, which has been extirpated from here and throughout the vast majority of its range.

### Tree species used in the experiments

We used seeds from five native tree species that included a fleshy fruited vertebrate-dispersed species (*Dimocarpus longan*; Sapindaceae) and four Dipterocarpaceae species that are non-fleshy and varied in size from 0.37 to 11.75 g (Fig. 2). *Dimocarpus longan* is a 10–25 m tall tree, cultivated for its drupaceous fruits that are similar to (and related to) lychee, which grows natively in Bornean forests^64^. All four species of dipterocarps are tall trees as adults (up to 45–70 m) and produce acorn-like nuts with 3–5 wings to aid in wind dispersal (Fig. 2)^64^. We chose these species because they represented a wide range of seed sizes, included both fleshy and non-fleshy fruits, and were widely available during the mast year in which we worked. Dipterocarp seeds germinate within ~30 days and die if they fail to germinate^65^; *Dimocarpus longan* seeds germinate after an average of ~21 days^66^, dying if they fail to germinate after 7 weeks^67^. We obtained dipterocarp seeds during a mast-fruiting event in August 2019. *Dimocarpus* seeds were purchased from nearby markets. As we were interested in seed predation rather than seed dispersal, we removed the *Dimocarpus* fruit flesh by hand, using the round, dark-colored seeds in our experiments.

### Nested exclusion experiment design

We established 10 replicate experimental blocks, each consisting of a 9 × 11 m, open-top exclosure with 1.8 m tall fencing of 4 × 4 cm wire mesh. These exclosures were designed to exclude large terrestrial vertebrates (>1 kg) such as elephants, bearded pigs, deer, and porcupines, but not smaller rodents or arboreal species such as primates, squirrels, or birds. Exclosures were spaced 75 m apart along a transect adjacent to the 50 ha permanent forest dynamics plot. We established six treatments per species within each of the 10 blocks (Fig. 1). For treatment 1 (control), seeds were placed outside the exclosure, accessible to all seed predators. For treatments 2–6, seeds were placed inside the exclosure, excluding large vertebrate seed predators. For treatments 3–6, seeds were protected by small, closed-top 1.3 cm wire mesh rodent exclosures. For treatments 4 and 6, seeds were sprayed with a broad-spectrum insecticide (malathion) and another insecticide that proved to be more effective at stopping ants (chlorpyrifos). We prepared concentrations of these insecticides following product instructions (2.5 mL 57% w/w malathion per 1 L of water, and 23.5 mL 21.2% w/w chlorpyrifos per 10 L of water). For treatments 5 and 6, seeds were sprayed with a broad-spectrum fungicide (mancozeb) at the company-specified concentration of 2.5 g 80.0% w/w mancozeb per 1 L of water. Thus, treatment 4 was only treated with insecticide, treatment 5 was only treated with fungicide, and treatment 6 was treated with both insecticide and fungicide. Both insecticides and fungicide were applied twice per week, and we sprayed an equivalent volume of water on other treatments to reduce bias associated with repeatedly visiting sites.

We opted for this nested design, rather than a fully factorial experimental design, for two reasons. First, we did not want to expose vertebrates to potentially harmful insecticides and fungicides. Second, we wanted our experimental design to mimic real-world patterns of defaunation, where large vertebrates are lost while insects and fungi remain, while still isolating the impacts of different groups of seed predators. Experimental treatments with large vertebrates but without insects or fungi (which would be part of a factorial design) are not ecologically realistic.

### Seed fate measurements

In each treatment in each block, we placed 10 seeds of each species within a 30 cm diameter circle, excluding seeds that showed preexisting visible damage. In total, our experiment included 600 seeds per species and 3000 seeds total. Every week we recorded the number of seeds that survived, the number that germinated, and the number of seedlings that established. Seeds germinated when the radicle emerged and were considered to have established when the cotyledons unfurled.

We monitored seeds for 11 weeks, at which point all seeds were either established or assumed to be dead. Given that our species all have short germination times and do not remain viable for long, we are confident that any seeds that had not germinated by the end of the study would never have done so. We assessed how many seeds died before they could germinate, how many germinated but died before they could establish, and how many successfully established. For seeds that died, we attributed mortality to either vertebrate predation or non-vertebrate mortality. Mortality was attributed to vertebrates either based on chewed seeds and tooth marks, or if seeds disappeared. Dipterocarp species were much more likely to be consumed by vertebrates in-place rather than to be removed, and the former left obvious remains. For *Dryobalanops lanceolata*, *Parashorea malaanonan*, and *Shorea leprosula*, we tagged half of the seeds in each vertebrate-accessible treatment by tying 1-meter-long strings around each tagged seed. We monitored these tagged seeds for the first five weeks of the 11-week study. During the first five weeks, we observed very few moved seeds (6 out of 300; Supplementary Table 1). After week 5, we continued to record seed fate for all seeds, but we did not specifically monitor tagged seeds, so we do not have a record of whether tagged seeds were moved during that period. Unlike dipterocarp seeds, *Dimocarpus longan* seeds were moved frequently, particularly in treatments accessible to large vertebrates (treatment 1). It is possible that these seeds may have dispersed intact rather than destroyed, but because far fewer seeds disappeared in treatment 2, the seeds in the control treatment were likely to have been consumed by large vertebrates rather than dispersed by scatter-hoarding rodents. Still, we may have overestimated vertebrate predation of *Dimocarpus longan*. We could not confidently differentiate mortality caused by insects or fungi specifically, so we classified all other mortality as non-vertebrate mortality.

### Logistic mixed-effects regression

We used mixed-effect logistic regressions to assess the probability that any given seed was (a) killed by vertebrates or (b) survived to a given stage. For all regressions described below, we included block as a random effect (ten levels). Analyses were performed using the lme4 package^68^ version 1.1–19 in R^69^ version 3.5.1.

In the model assessing seed mortality caused by vertebrates, the dataset was limited to treatments 1 and 2, since these were the only two treatments accessible to vertebrates. In this model, we also excluded the species *Shorea macrophylla*, as this species had no seeds predated by vertebrates in treatment 2. Our predictor variables were treatment (a fixed effect with two levels for the open controls versus inside fences) and seed-stage (a fixed effect with two levels: germinated or established). We included species as a random effect (four levels, one for each species included in the analysis), with random intercepts and random slopes. This allowed us to determine whether the effects of treatment were consistent across species.

To assess species-specific responses to treatments, we also ran separate models for each of the four species (again excluding *Shorea macrophylla*). For each of these models our predictor variables were treatment (a fixed effect with two levels for the open controls versus inside fences) and seed-stage (a fixed effect with two levels: germinated or established).

In the regression assessing seed survival, the dataset included all six treatments and all five species. Our predictor variables were treatment (a fixed effect with six levels for the six treatments, see Fig. 1) and seed stage (a fixed effect with two levels: germinated or established). We included species as a random effect (five levels, one for each species), with random intercepts and random slopes. To test for differences among treatments, we conducted post-hoc Tukey tests to adjust for multiple comparisons, using the multicomp package^70^ version 1.4–15. We used a subset of the pairwise comparisons from the post-hoc tests to identify particular exclusion effects on seed survival: treatments 1 vs. 2 for the effect of large vertebrate exclosure, 2 vs. 3 for small vertebrate exclosure, 3 vs. 4 for insecticide, 3 vs. 5 for fungicide, and 3 vs. 6 for the combined effects of insecticide and fungicide. We also ran species-specific models with treatment (a fixed effect with six levels for the six treatments) and seed-stage (a fixed effect with two levels: germinated or established) as predictor variables. To test for differences among treatments, we again conducted post-hoc Tukey tests. We used the same subsets of pairwise comparisons listed above to identify particular exclusion effects on seed survival.

The full results from all regressions, including all pairwise comparisons from post-hoc tests, are presented in the supplemental material (Supplementary Tables 2–8).

### Reporting summary

Further information on research design is available in the Nature Research Reporting Summary linked to this article.

## Supplementary information

Supplementary Information

Reporting Summary

## Data Availability

Data are available at https://figshare.com/articles/dataset/Williams_NatComm2021_seed_predation_data_csv/13699087.

## References

[1] Dirzo R (2014). Defaunation in the anthropocene. Science.

[2] Harrison RD (2016). Impacts of hunting on tropical forests in Southeast Asia. Conserv. Biol..

[3] Brodie JF, Aslan CE (2012). Halting regime shifts in floristically intact tropical forests deprived of their frugivores. Restor. Ecol..

[4] Bello C (2015). Defaunation affects carbon storage in tropical forests. Sci. Adv..

[5] Gardner CJ, Bicknell JE, Baldwin-Cantello W, Struebig MJ, Davies ZG (2019). Quantifying the impacts of defaunation on natural forest regeneration in a global meta-analysis. Nat. Commun..

[6] Osuri AM (2016). Contrasting effects of defaunation on aboveground carbon storage across the global tropics. Nat. Commun..

[7] Peres CA, Emilio T, Schietti J, Desmoulière SJM, Levi T (2016). Dispersal limitation induces long-term biomass collapse in overhunted Amazonian forests. Proc. Natl Acad. Sci. USA.

[8] Dantas de Paula M (2018). Defaunation impacts on seed survival and its effect on the biomass of future tropical forests. Oikos.

[9] Chanthorn W (2019). Defaunation of large-bodied frugivores reduces carbon storage in a tropical forest of Southeast Asia. Sci. Rep..

[10] Comita LS (2014). Testing predictions of the Janzen-Connell hypothesis: a meta-analysis of experimental evidence for distance- and density-dependent seed and seedling survival. J. Ecol..

[11] Song X, Lim JY, Yang J, Luskin MS (2020). When do Janzen–Connell effects matter? A phylogenetic meta‐analysis of conspecific negative distance and density dependence experiments. Ecol. Lett..

[12] Muller-Landau HC (2007). Predicting the long-term effects of hunting on plant species composition and diversity in tropical forests. Biotropica.

[13] Asquith NM, Wright SJ, Clauss MJ (1997). Does mammal community composition control recruitment in neotropical forests? Evidence from Panama. Ecology.

[14] DeMattia EA, Curran LM, Rathcke BJ (2004). Effects of small rodents and large mammals on neotropical seeds. Ecology.

[15] Paine CET, Beck H, Terborgh J (2016). How mammalian predation contributes to tropical tree community structure. Ecology.

[16] Wirth, R., Meyer, S. T., Leal, I. R. & Tabarelli, M. Plant herbivore interactions at the forest edge. in *Progress in Botany* (eds. Lüttge, U., Beyschlag, W. & Murata, J.). **69**, 423–448 (Springer, Berlin, Heidelberg, 2008).

[17] Paine CET, Beck H (2007). Seed predation by Neotropical rain forest mammals increases diversity in seedling recruitment. Ecology.

[18] Jia S (2018). Global signal of top-down control of terrestrial plant communities by herbivores. Proc. Natl Acad. Sci. USA.

[19] Wright SJ, Duber HC (2001). Poachers and forest fragmentation alter seed dispersal, seed survival, and seedling recruitment in the palm Attalea butyraceae, with implications for tropical tree diversity. Biotropica.

[20] Dracxler CM, Pires AS, Fernandez FAS (2011). Invertebrate seed predators are not all the same: Seed predation by bruchine and scolytine beetles affects palm recruitment in different ways. Biotropica.

[21] Sarmiento C (2017). Soilborne fungi have host affinity and host-specific effects on seed germination and survival in a lowland tropical forest. Proc. Natl Acad. Sci..

[22] Kluger CG (2008). Host generalists dominate fungal communities associated with seeds of four Neotropical pioneer species. J. Trop. Ecol..

[23] Velho N, Isvaran K, Datta A (2012). Rodent seed predation: effects on seed survival, recruitment, abundance, and dispersion of bird-dispersed tropical trees. Oecologia.

[24] Curran LM, Webb CO (2000). Experimental tests of the spatiotemporal scale of seed predation in mast-fruiting Dipterocarpaceae. Ecol. Monogr..

[25] Janzen DH (1970). Herbivores and the number of tree species in tropical forests. Am. Nat..

[26] Connell JH (1971). On the role of natural enemies in preventing competitive exclusion in some marine animals and in rain forest trees. Dyn. Popul.

[27] Levi T (2019). Tropical forests can maintain hyperdiversity because of enemies. PNAS.

[28] Terborgh J (2012). Enemies maintain hyperdiverse tropical forests. Am. Nat..

[29] Nathan R, Casagrandi R (2004). A simple mechanistic model of seed dispersal, predation and plant establishment: Janzen-Connell and beyond. J. Ecol..

[30] Owen-Smith, R. N. *Megaherbivores: The influence of very large body size on ecology*. (Cambridge University Press, 1988).

[31] Kurten EL (2013). Cascading effects of contemporaneous defaunation on tropical forest communities. Biol. Conserv..

[32] Mendoza E, Dirzo R (2007). Seed-size variation determines interspecific differential predation by mammals in a Neotropical rain forest. Oikos.

[33] Wright SJ (2003). The myriad consequences of hunting for vertebrates and plants in tropical forests. Perspect. Plant Ecol. Evol. Syst..

[34] Casula P, Wilby A, Thomas MB (2006). Understanding biodiversity effects on prey in multi-enemy systems. Ecol. Lett..

[35] Wright SJ (2000). Poachers alter mammal abundance, seed dispersal, and seed predation in a Neotropical forest. Conserv. Biol..

[36] Beckman NG, Muller-landau HC (2007). Differential effects of hunting on pre-dispersal seed predation and primary and secondary seed removal of two Neotropical tree species. Biotropica.

[37] Harrison RD (2013). Consequences of defaunation for a tropical tree community. Ecol. Lett..

[38] Galetti M, Bovendorp RS, Guevara R (2015). Defaunation of large mammals leads to an increase in seed predation in the Atlantic forests. Glob. Ecol. Conserv.

[39] Culot L, Bello C, Batista JLF, do Couto HTZ, Galetti M (2017). Synergistic effects of seed disperser and predator loss on recruitment success and long-term consequences for carbon stocks in tropical rainforests. Sci. Rep..

[40] Rosin C, Poulsen JR (2016). Hunting-induced defaunation drives increased seed predation and decreased seedling establishment of commercially important tree species in an Afrotropical forest. Ecol. Manag..

[41] Ceballos G, Ehrlich PR, Dirzo R (2017). Biological annihilation via the ongoing sixth mass extinction signaled by vertebrate population losses and declines. Proc. Natl Acad. Sci. USA.

[42] Terborgh J (2013). Using Janzen-Connell to predict the consequences of defaunation and other disturbances of tropical forests. Biol. Conserv..

[43] Brodie JF, Helmy OE, Brockelman WY, Maron JL (2009). Bushmeat poaching reduces the seed dispersal and population growth rate of a mammal-dispersed tree. Ecol. Appl..

[44] Dylewski L, Ortega YK, Bogdziewicz M, Pearson DE (2020). Seed size predicts global effects of small mammal seed predation on plant recruitment. Ecol. Lett..

[45] Bodmer R (1991). Strategies of seed dispersal and seed predation in Amazonian ungulates. Biotropica.

[46] Galetti M (2015). Defaunation affect population and diet of rodents in Neotropical rainforests. Biol. Conserv..

[47] Dirzo R, Mendoza E, Ortíz P (2007). Size-related differential seed predation in a heavily defaunated neotropical rain forest. Biotropica.

[48] Luskin MS (2017). Cross-boundary subsidy cascades from oil palm degrade distant tropical forests. Nat. Commun..

[49] Vázquez-Yanes C, Orozco-Segovia A (1993). Patterns of seed longevity and germination in the tropical rainforest. Annu. Rev. Ecol. Syst..

[50] Hulme PE (1998). Post-dispersal seed predation and seed bank persistence. Seed Sci. Res..

[51] Franco M, Silvertown J (2004). A comparative demography of plants based upon elasticities of vital rates. Ecology.

[52] Howe HF, Miriti MN (2004). When seed dispersal matters. Bioscience.

[53] Cannon PG, O’Brien MJ, Yusah KM, Edwards DP, Freckleton RP (2020). Limited contributions of plant pathogens to density-dependent seedling mortality of mast fruiting Bornean trees. Ecol. Evol..

[54] Lamperty T, Zhu K, Poulsen JR, Dunham AE (2020). Defaunation of large mammals alters understory vegetation and functional importance of invertebrates in an Afrotropical forest. Biol. Conserv..

[55] Ewers RM (2015). Logging cuts the functional importance of invertebrates in tropical rainforest. Nat. Commun..

[56] Peguero G, Muller-Landau HC, Jansen PA, Wright SJ (2017). Cascading effects of defaunation on the coexistence of two specialized insect seed predators. J. Anim. Ecol..

[57] Marsh CW, Greer AG (1992). Forest land-use in Sabah, Malaysia: an introduction to danum valley. Philos. Trans. R. Soc. B Biol. Sci..

[58] Dial R, Bloodworth B, Lee A, Boyne P, Heys J (2004). The distribution of free space and its relation to canopy composition at six forest sites. Science.

[59] Sakai S (2002). General flowering in lowland mixed dipterocarp forests of South-east Asia. *Biol*. J. Linn. Soc..

[60] Blate GM, Peart DR, Leighton M (1998). Post-dispersal predation on isolated seeds: a comparative study of 40 tree species in a Southeast Asian rainforest. Oikos.

[61] Wong STE, Servheen C, Ambu L, Norhayati A (2005). Impacts of fruit production cycles on Malayan sun bears and bearded pigs in lowland tropical forest of Sabah, Malaysian Borneo. J. Trop. Ecol..

[62] Curran LM, Leighton M (2000). Vertebrate responses to spatiotemporal variation in seed production of mast-fruiting Dipterocarpaceae. Ecol. Monogr..

[63] Corlett RT (2017). Frugivory and seed dispersal by vertebrates in tropical and subtropical Asia: an update. Glob. Ecol. Conserv..

[64] Fern, K. Tropical Plants Database. (2014). Available at: tropical.theferns.info. (Accessed: 4th June 2020)

[65] O’Brien MJ, Philipson CD, Tay J, Hector A (2013). The influence of variable rainfall frequency on germination and early growth of shade-tolerant dipterocarp seedlings in Borneo. PLoS ONE.

[66] Colon CP, Campos-Arceiz A (2013). The impact of gut passage by binturongs (Arctictus binturong) on seed germination. Raffles Bull. Zool..

[67] Sowa S, Roos EE, Zee F (1991). Anesthetic storage of recalcitrant seed: nitrous oxide prolongs longevity of lychee and longan. HortScience.

[68] Bates D, Mächler M, Bolker BM, Walker SC (2015). Fitting linear mixed-effects models using lme4. J. Stat. Softw..

[69] R Core Team. R: A language and environment for statistical computing. (2018).

[70] Hothorn T, Bretz F, Westfall P (2008). Simultaneous inference in general parametric models. Biometrical J..

