# Supplementary material for: Fungi and insects compensate for lost vertebrate seed predation in an experimentally defaunated tropical forest
Source: Nat Commun. 2021 Mar 12;12:1650. doi: 10.1038/s41467-021-21978-8 (PMC7955059; doi:10.1038/s41467-021-21978-8)
Supplement: Supplementary file 1 — Supplementary Information [file 41467_2021_21978_MOESM1_ESM.pdf]

Supplementary Information

Fungi and insects compensate for lost vertebrate seed predation in an experimentally defaunated tropical forest

Peter Jeffrey Williams[1]*, Robert C. Ong[2], **Jedediah F. Brodie[1,3], **Matthew Scott Luskin[4]

[1]Division of Biological Sciences, University of Montana, 32 Campus Drive, Missoula MT 59812, USA

[2]Sabah Forestry Department, Forest Research Centre, Sepilok, P.O. Box 1407, 90715 Sandakan, Sabah, Malaysia

[3]Wildlife Biology Program, University of Montana, 32 Campus Drive, Missoula MT 59812, USA

[4]School of Biological Sciences, University of Queensland, 534 Goddard Hall, St. Lucia, QLD, Australia

*Correspondence and requests for materials should be addressed to P.J.W. (email: peter.j.williams.110@gmail.com)

**These authors jointly supervised this work

SUPPLEMENTARY TABLES

Supplementary Table 1. Tagged seeds to monitor secondary dispersal. We tagged half of all seeds in vertebrate-accessible treatments, 5 of 10 seeds per species per treatment per block. We tied 1-meter-long string around seeds to tag them. Tagged seeds were monitored for the first 5 weeks, though the study lasted 11 weeks. From weeks 5 to 11, seed fate was recorded for all seeds including tagged seeds, but tagged seeds were not specifically monitored those weeks, so we do not have a record of whether tagged seeds were moved after week 5. Percentages are based on the number of tagged seeds (100 per species) or the number of total seeds in vertebrate-accessible treatments (200 per species).

Species	% tagged seeds moved	% tagged seeds moved and intact	% total seeds predated by end of monitoring period (first 5 weeks)	% total seeds predated by end of study (11 weeks)
Dryobalanops lanceolata	4	1	11	15
Parashorea malaanonan	0	0	0	4.5
Shorea leprosula	2	2	0	8

Supplementary Table 2. Vertebrate-caused morality logistic regressions, two-tailed. Each model compares the difference in vertebrate predation between treatment 1 (accessible to all vertebrates) and treatment 2 (only accessible to small vertebrates). Methods described in main text. We did not include *Shorea macrophylla* in the overall model, nor did we run an individual species model on *Shorea macrophylla*, because this species had no seeds predated by vertebrates in treatment 2. Significant *P*-values, not adjusted for multiple comparisons, are in bold, * $P < .01$, ** $P < .001$.

Model	β ± SE	*P*
All species (‘species’ as random effect)	-1.89 ± 0.47	**<.001****
Dimocarpus longan	-6.51 ± 0.85	**<.001****
Dryobalanops lanceolata	-1.35 ± 0.41	**<.001****
Parashorea malaanonan	-1.51 ± 0.87	.083
Shorea leprosula	-2.21 ± 0.66	**<.001****

Supplementary Table 3. Pairwise comparisons for seed survival logistic regression, two-tailed, all species. This model included data from all five seed species, with *species* included as a random effect. Methods described in main text. Values are from post-hoc two-tailed Tukey tests, $\beta \pm$ SE above the diagonal (upper right), *P*-values below the diagonal (lower left). Significant pairwise comparisons are in bold; * $P < .01$; ** $P < .001$.

Treatment	**1**	**2**	**3**	**4**	**5**	**6**
**1**	–	0.30 ± 0.30	0.67 ± 0.25	1.21 ± 0.33	0.97 ± 0.41	1.16 ± 0.36
**2**	.887	–	0.37 ± 0.21	0.91 ± 0.27	0.67 ± 0.21	0.86 ± 0.19
**3**	.054	.411	–	0.53 ± 0.16	0.30 ± 0.32	0.49 ± 0.19
**4**	**.003***	**.007***	**.011**	–	-0.24 ± 0.37	-0.04 ± 0.19
**5**	.137	**.011**	.902	.981	–	0.19 ± 0.28
**6**	**.013**	**<.001****	.086	1.000	.972	–

Supplementary Table 4. Pairwise comparisons for seed survival logistic regression, two-tailed, *Dimocarpus longan*. This model only included data for this single species. Methods described in main text. Values are from post-hoc two-tailed Tukey tests, β ± SE above the diagonal (upper right), P-values below the diagonal (lower left). Significant pairwise comparisons are in bold; * $P < .01$; ** $P < .001$.

Treatment	**1**	**2**	**3**	**4**	**5**	**6**
**1**	–	1.70 ± 0.36	1.82 ± 0.36	2.89 ± 0.37	2.85 ± 0.37	3.29 ± 0.37
**2**	**<.001****	–	0.12 ± 0.28	1.19 ± 0.28	1.15 ± 0.28	1.58 ± 0.29
**3**	**<.001****	.998	–	1.07 ± 0.28	1.03 ± 0.28	1.46 ± 0.29
**4**	**<.001****	**<.001****	**.002***	–	-0.04 ± 0.28	0.39 ± 0.28
**5**	**<.001****	**.001***	**.003***	1.000	–	0.43 ± 0.28
**6**	**<.001****	**<.001****	**<.001****	.724	.637	–

Supplementary Table 5. Pairwise comparisons for seed survival logistic regression, two-tailed, *Dryobalanops lanceolata*. This model only included data for this single species. Methods described in main text. Values are from post-hoc two-tailed Tukey tests, β ± SE above the diagonal (upper right), P-values below the diagonal (lower left). Significant pairwise comparisons are in bold; * $P < .01$; ** $P < .001$.

Treatment	Treatment: 1	2	3	4	5	6
**1**	–	0.11 ± 0.27	0.69 ± 0.28	1.42 ± 0.31	0.26 ± 0.27	1.05 ± 0.29
**2**	.999	–	0.58 ± 0.28	1.31 ± 0.31	0.15 ± 0.27	0.94 ± 0.29
**3**	.132	.297	–	0.73 ± 0.32	-0.44 ± 0.28	0.36 ± 0.30
**4**	**<.001****	**<.001****	.191	–	-1.16 ± 0.31	-0.37 ± 0.33
**5**	.934	.994	.635	**.002***	–	0.80 ± 0.29
**6**	**.004***	**.016**	.837	.867	.073	–

Supplementary Table 6. Pairwise comparisons for seed survival logistic regression, two-tailed, *Parashorea malaanonan*. This model only included data for this single species. Methods described in main text. Values are from post-hoc two-tailed Tukey tests, β ± SE above the diagonal (upper right), P-values below the diagonal (lower left). Significant pairwise comparisons are in bold; * $P < .01$; ** $P < .001$.

Treatment		Treatment					
		**1**	**2**	**3**	**4**	**5**	**6**
	**1**	–	-0.64 ± 0.28	0.87 ± 0.31	1.67 ± 0.35	0.35 ± 0.30	0.99 ± 0.32
	**2**	.182	–	1.51 ± 0.31	2.31 ± 0.35	0.99 ± 0.29	1.63 ± 0.31
	**3**	.063	**<.001****	–	0.80 ± 0.37	-0.52 ± 0.32	0.12 ± 0.34
	**4**	**<.001****	**<.001****	.247	–	-1.32 ± 0.36	-0.69 ± 0.37
	**5**	.839	**.007***	.590	**.003***	–	0.63 ± 0.33
	**6**	**.024**	**<.001****	.999	.435	.372	–

Supplementary Table 7. Pairwise comparisons for seed survival logistic regression, two-tailed, *Shorea leprosula*. This model only included data for this single species. Methods described in main text. Values are from post-hoc two-tailed Tukey tests, $\beta \pm$ SE above the diagonal (upper right), P-values below the diagonal (lower left). Significant pairwise comparisons are in bold; * $P < .01$; ** $P < .001$.

		Treatment					
		**1**	**2**	**3**	**4**	**5**	**6**
**Treatment**	**1**	–	0.40 ± 0.25	0.37 ± 0.25	0.50 ± 0.25	1.76 ± 0.30	0.74 ± 0.26
	**2**	.590	–	-0.03 ± 0.25	0.10 ± 0.26	1.36 ± 0.31	0.34 ± 0.26
	**3**	.673	1.000	–	0.13 ± 0.26	1.39 ± 0.31	0.38 ± 0.26
	**4**	.346	.999	.996	–	1.26 ± 0.31	0.25 ± 0.26
	**5**	**<.001****	**<.001****	**<.001****	**<.001****	–	-1.01 ± 0.31
	**6**	**.044**	.777	.702	.939	**.015**	–

Supplementary Table 8. Pairwise comparisons for seed survival logistic regression, two-tailed, *Shorea macrophylla*. This model only included data for this single species. Methods described in main text. Values are from post-hoc two-tailed Tukey tests, β ± SE above the diagonal (upper right), *P*-values below the diagonal (lower left). Significant pairwise comparisons are in bold; * $P < .01$; ** $P < .001$.

Treatment		**1**	**2**	**3**	**4**	**5**	**6**
Treatment	**1**	–	0.03 ± 0.26	0.10 ± 0.26	0.47 ± 0.25	0.20 ± 0.26	0.55 ± 0.25
	**2**	1.000	–	0.07 ± 0.26	0.43 ± 0.25	0.17 ± 0.26	0.52 ± 0.25
	**3**	.999	1.000	–	0.37 ± 0.25	0.10 ± 0.25	0.45 ± 0.25
	**4**	.425	.509	.679	–	-0.27 ± 0.24	0.08 ± 0.23
	**5**	.972	.988	.999	.882	–	0.35 ± 0.24
	**6**	.234	.298	.449	.999	.697	–

SUPPLEMENTARY FIGURES

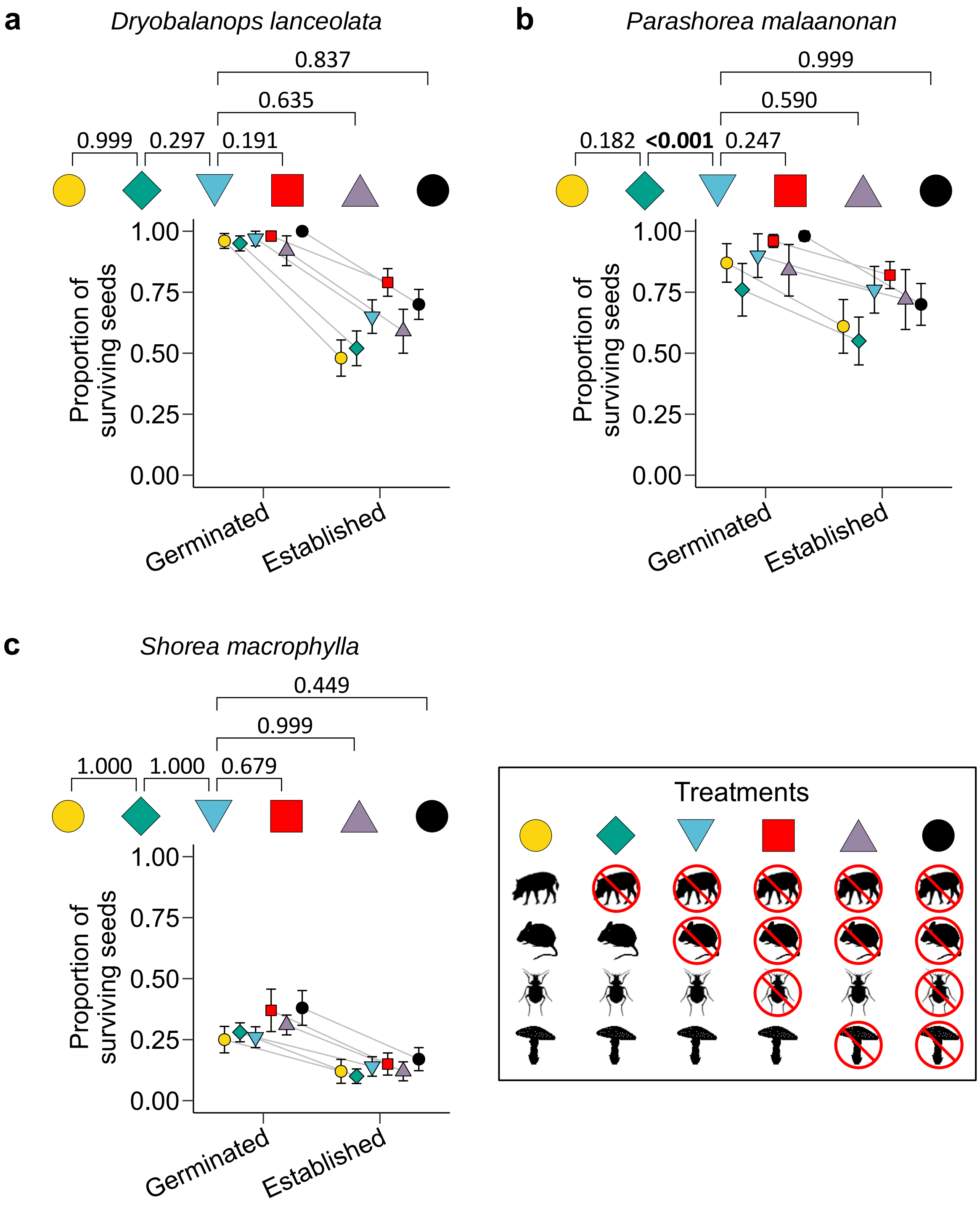

Supplementary Figure 1: Proportions of seeds surviving under experimental treatments (indicated by different colors and shapes); error bars indicate mean values ± standard errors

across treatments (N = 50 for all species; N = 10 for individual species). Logistic regressions were performed to test the effect of treatment on seed survival. Then, post-hoc Tukey tests were used to adjust for multiple tests and identify significant pairwise comparisons. All statistical tests were two-tailed. Selected pairwise comparisons shown with *P*-values; significant comparisons in bold.
