# Supplementary material for: Fungi and insects compensate for lost vertebrate seed predation in an experimentally defaunated tropical forest
Source: Nat Commun. 2021 Mar 12;12:1650. doi: 10.1038/s41467-021-21978-8 (PMC7955059; doi:10.1038/s41467-021-21978-8)
Supplement: Supplementary file 2 — Reporting Summary [file 41467_2021_21978_MOESM2_ESM.pdf]

nature research

Corresponding author(s): Peter Jeffrey Williams

Last updated by author(s): Feb 3, 2021

Reporting Summary

Nature Research wishes to improve the reproducibility of the work that we publish. This form provides structure for consistency and transparency in reporting. For further information on Nature Research policies, see our Editorial Policies and the Editorial Policy Checklist.

Statistics

For all statistical analyses, confirm that the following items are present in the figure legend, table legend, main text, or Methods section.

n/a	Confirmed	
☐	☒	The exact sample size (n) for each experimental group/condition, given as a discrete number and unit of measurement
☐	☒	A statement on whether measurements were taken from distinct samples or whether the same sample was measured repeatedly
☐	☒	The statistical test(s) used AND whether they are one- or two-sided<br>*Only common tests should be described solely by name; describe more complex techniques in the Methods section.*
☐	☒	A description of all covariates tested
☐	☒	A description of any assumptions or corrections, such as tests of normality and adjustment for multiple comparisons
☐	☒	A full description of the statistical parameters including central tendency (e.g. means) or other basic estimates (e.g. regression coefficient) AND variation (e.g. standard deviation) or associated estimates of uncertainty (e.g. confidence intervals)
☐	☒	For null hypothesis testing, the test statistic (e.g. F, t, r) with confidence intervals, effect sizes, degrees of freedom and P value noted<br>*Give P values as exact values whenever suitable.*
☒	☐	For Bayesian analysis, information on the choice of priors and Markov chain Monte Carlo settings
☒	☐	For hierarchical and complex designs, identification of the appropriate level for tests and full reporting of outcomes
☒	☐	Estimates of effect sizes (e.g. Cohen's d, Pearson's r), indicating how they were calculated

Our web collection on statistics for biologists contains articles on many of the points above.

Software and code

Policy information about availability of computer code

Data collection	Data were manually entered into a spreadsheet, but no software was used to collect data.
Data analysis	Analyses were performed using the lme4 package version 1.1-19 and the multcomp package version 1.4-15 in R version 3.5.1.

For manuscripts utilizing custom algorithms or software that are central to the research but not yet described in published literature, software must be made available to editors and reviewers. We strongly encourage code deposition in a community repository (e.g. GitHub). See the Nature Research guidelines for submitting code & software for further information.

Data

Policy information about availability of data

All manuscripts must include a data availability statement. This statement should provide the following information, where applicable:

- Accession codes, unique identifiers, or web links for publicly available datasets
- A list of figures that have associated raw data
- A description of any restrictions on data availability

Data are available at https://figshare.com/articles/dataset/Williams_NatComm2021_seed_predation_data_csv/13699087

Field-specific reporting

Please select the one below that is the best fit for your research. If you are not sure, read the appropriate sections before making your selection.

☐ Life sciences ☐ Behavioural & social sciences ☒ Ecological, evolutionary & environmental sciences

For a reference copy of the document with all sections, see nature.com/documents/nr-reporting-summary-flat.pdf

Ecological, evolutionary & environmental sciences study design

All studies must disclose on these points even when the disclosure is negative.

Study description	Nested exclusion seed mortality experiment. 10 replicate blocks, each block containing the same 6 experimental treatments. The treatments consisted of: 1) nothing excluded; 2) large vertebrates excluded; 3) all vertebrates excluded; 4) insects and all vertebrates excluded; 5) fungi and all vertebrates excluded; and 6) insects, fungi, and all vertebrates excluded. The same 10 blocks and 6 treatments used for all five tree species. 10 seeds of each species were placed in each treatment of each block.
Research sample	Seeds from 5 native tree species: Dimocarpus longan, Dryobalanops lanceolata, Parashorea malaanonan, Shorea leprosula, and Shorea macrophylla. These species were chosen because they represented a wide range of seed sizes, included both fleshy and non-fleshy fruits, and were widely available during the mast year in which we worked.
Sampling strategy	Seeds for 4 species were collected from fruiting trees, either picking seed off the ground or using a large slingshot to shake seeds off of high branches. For Dimocarpus logan, we bought longan fruits in the local market and collected seeds after consuming the flesh of the fruits. We removed seeds that showed pre-existing damage. We placing seeds in the experimental treatments, we randomly selected seeds from the total collection of seeds.
Data collection	Seeds were monitored by P. Williams, J. Suffian, N. Thomas, M. Markus, A. Karolus, and F. Karolus. We visually observed seeds, and noted seed fate on a printed datasheet, which was later compiled as a spreadsheet.
Timing and spatial scale	Data was collected for 11 weeks, from mid-August to late October 2019, after which point all seeds either established as seedlings or died. Given that all five species have short germination times and lack seedbanks, it is very unlikely that any seeds that had failed to establish by the end of the study were still viable. We began our experiment in late 2019 because that was the only time seeds were naturally available in the forest due to the mast fruiting event. Data for germination, seedling establishments, and vertebrate mortality were recorded once a week, which we deemed sufficient for observed changes in seed fate. The spatial scale of data collection covered a 675m transect in a single forest. Along the transect, 10 large-vertebrate exclosure sites were spaced 75 m apart from one another.
Data exclusions	Seeds showing preexisting visible damage were not placed in experimental treatments, but all seeds placed in treatments were included in analyses.
Reproducibility	The experiment was replicated at 10 blocks along a transect, each block containing all combinations of species and treatments. All 10 replicates were successful. Data from all 10 replicates is included in the manuscript.
Randomization	Seeds were randomly collected, and seeds were randomly drawn when placing seeds in experimental treatments.
Blinding	We did not use blinding in our study. We studied whether organisms killed our seeds, and these organisms would not be biased toward achieving certain results. As for observing seed fate, we chose to focus on the emergence of the radicle (germination) and the unfurling of cotyledons (seedling establishment), as these stages were less subjective than identifying whether a seed had truly died or was merely late to germinate. Due to logistics and lack of resources, we had to rely on research assistants to visual observe seed fate in rather than blinding the observers.

Did the study involve field work? ☒ Yes ☐ No

Field work, collection and transport

Field conditions	Primary lowland dipterocarp forest; ~2800 mm annual rainfall; temperature range generally 23-31°C
Location	Danum Valley Field Center, Sabah, Malaysia; N 4.96° E 117.80°
Access & import/export	We received permission from Yayasan Sabah, the Sabah Forest Department, the Sabah Biodiversity Council, and the Danum Valley Management Committee to conduct research at the field site. We did not import or export any samples.
Disturbance	Stayed on previously established trails. Only sprayed insecticide and fungicide in treatments inaccessible to vertebrates.

Reporting for specific materials, systems and methods

We require information from authors about some types of materials, experimental systems and methods used in many studies. Here, indicate whether each material, system or method listed is relevant to your study. If you are not sure if a list item applies to your research, read the appropriate section before selecting a response.

Materials & experimental systems

n/a	Involved in the study
☒	☐ Antibodies
☒	☐ Eukaryotic cell lines
☒	☐ Palaeontology and archaeology
☒	☐ Animals and other organisms
☒	☐ Human research participants
☒	☐ Clinical data
☒	☐ Dual use research of concern

Methods

n/a	Involved in the study
☒	☐ ChIP-seq
☒	☐ Flow cytometry
☒	☐ MRI-based neuroimaging
